# Coronary artery to pulmonary artery fistula: Catheter or scalpel? A case report

**DOI:** 10.1016/j.ijscr.2022.107416

**Published:** 2022-07-16

**Authors:** Mian Mustafa Kamal, Rita Sundardas, Abdul Ahad Sohail, Majid Usman, Sara Iqbal, Fateh Ali Tipu, Saulat Hasnain Fatimi

**Affiliations:** a Department of Cardiothoracic Surgery, Aga Khan University Hospital Karachi, Pakistan; b Department of Cardiology, Aga Khan University Hospital Karachi, Pakistan

**Keywords:** Case report, Congenital coronary artery fistula, Surgical closure, LAD to PA fistula, Coronary artery disease

## Abstract

**Introduction:**

Coronary artery fistula (CAF) is an abnormal connection between coronary artery and a major vessel or cardiac chamber with left to right shunt having an incidence of 0.002 % in recent literature. Fistulous communication of coronary artery with pulmonary artery (PA) is a rare subtype and comprises of about 17 % of all the CAF cases.

**Case presentation:**

We report a case of a middle-aged gentleman, known case of asymptomatic CAF for the last 20 years. He presented to us with 6 months history of chest pain on exertion. On coronory angiogram he was diagnosed to have a preexisting CAF of proximal LAD to main PA and severe coronary artery disease in left anterior descending coronary artery (LAD). He was managed surgically and underwent ligation of the fistula along with coronary artery bypass grafting (CABG).

**Clinical discussion:**

Management of CAF is medical, percutaneous or open-heart surgery. Due to rarity of the disease no international guidelines exists and treatment is controversial. Complications of CAF include endocarditis, early atherosclerosis, rupture, hemopericardium, pulmonary hypertension and myocardial ischemia, hence early correction is warranted. Our case emphasizes on the natural course of this rare disease and how to change management plan accordingly in the better interest of patient.

**Conclusion:**

Our case presents the natural course and management of a rare congenital cardiac disease. Surgery was chosen as an appropriate option due to CAD involving proximal LAD and concomitant coronary artery to PA fistula.

## Highlights

• Fistulous communication of coronary artery with pulmonary artery
• Can lead to myocardial ischemia
• Critical left anterior descending and left circumflex artery disease
• Surgical ligation of Fistula and Coronary Artery Bypass Graft

## 1 Introduction

Coronary artery fistula (CAF) is defined as an abnormal connection between coronary artery and a major vessel or cardiac chamber [1], [2]. Majority of the CAF are congenital and its incidence in the general population is estimated to be 0.002 % in recent literature [1], [2]. Fistulous communication of coronary artery with pulmonary artery (PA) is a rare subtype and comprises of about 17 % of the CAF cases [3]. CAF can occur as an isolated anomaly in 55–80 % of cases and associated with another congenital heart disease in 10–45 % of cases. Associated anomalies include atrial septal defect, tetralogy of Fallot, patent ductus arteriosus, ventricular septal defect and coarctation of the aorta. CAF were first described in 1865 and the first successful surgical closure of CAF was performed in 1947 [1]. The management of CAF is still controversial in terms of appropriate timing of intervention and invasive vs non-invasive approach. Due to rarity of disease no international guidelines exists and patients are managed on case-to-case basis. The patient was successfully managed in our private tertiary care hospital.

We report a case of a middle age gentleman, known case of CAF for the last 20 years and was asymptomatic on medical treatment. Now presented with 6 months history of angina pectoris, workup re confirmed diagnosis of CAF of proximal left anterior descending artery (LAD) to main PA in association with critical left anterior descending coronary artery disease. Due to CAF and CAD open surgical approach was planned, CAF was ligated epicardialy and coronary artery bypass grafting (CABG) was done to LAD. This case report has been reported in line with SCARE 2020 criteria [4].

## 2 Presentation of case

**Fig. 1:**
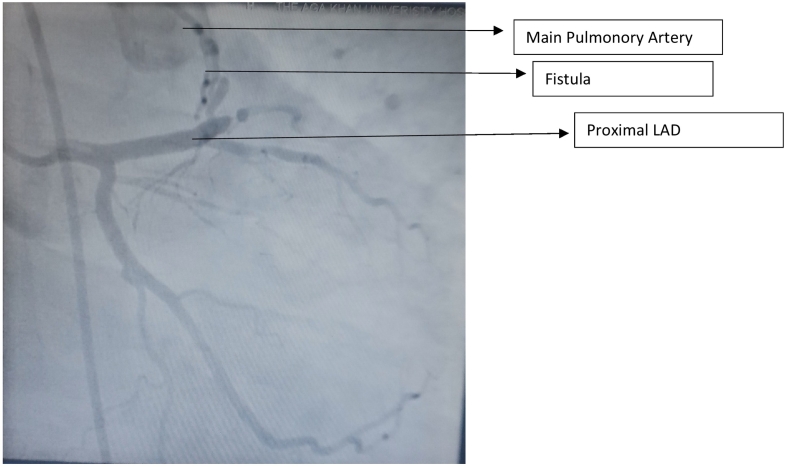
Coronary Angiography showing CAF arising from proximal LAD.

**Fig. 2:**
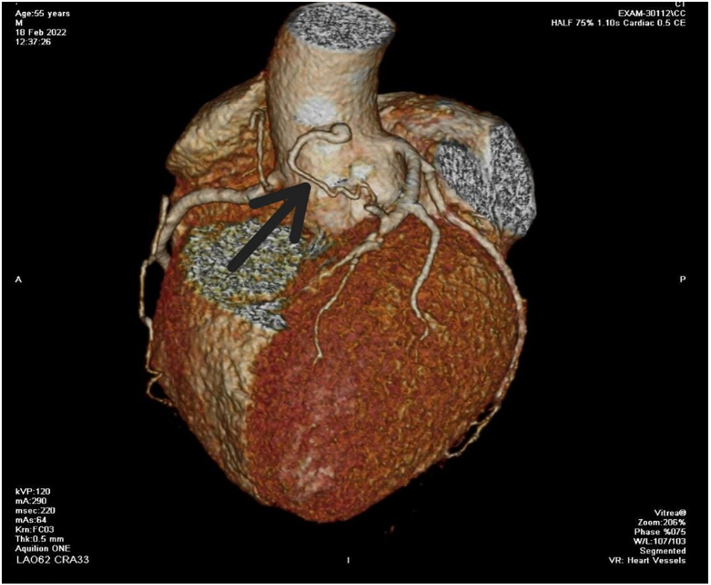
3D MDCT showing fistula (solid arrow) connecting LAD to PA.

We report a case of middle-aged gentleman, known case of hypertension and active cigarette smoker. No family history of acquired or congenital cardiac diseases. He was diagnosed with coronary artery to main pulmonary artery fistula in year 2002 when his left heart catheterization was done for atypical anginal symptoms, rest of coronaries were normal and hence he was advised conservative management for it. He had no significant past medical or surgical history. He remained asymptomatic during this period, but now presented to our cardiology outpatient department with 6 months history of chest pain on exertion. Angina severity was Canadian Cardiovascular Society Class III, radiating to back and was relieved with rest. Physical examination and cardiac auscultation were normal. Cardiac workup was done starting with 12 lead ECG and a chest X ray which were normal. Coronary angiography showed coronary artery fistula arising from the proximal LAD and draining into main PA (Fig. 1). Besides that, his angiography showed proximal LAD and 2nd diagonal having severe stenosis, however rest of the angiogram was normal. Right heart catheterization showed main PA pressure of 40/20 mmHg and no significant step up in oxygen saturation between right atrium (RA) and PA. Transthoracic ECHO was performed which showed left ventricular ejection fraction of 60 %, with normal valves and no wall motion abnormality. Computed tomography (CT) coronary angiography showed a single tortuous fistulous communication between proximal LAD and main PA. Proximal LAD had severe concentric stenosis however rest of the angiogram was normal (Fig. 2). As patient was symptomatic, surgical ligation of fistula along with coronary artery bypass graft surgery was planned because of severe LAD and Diagonal artery disease. Patient was admitted for elective coronary artery bypass graft (CABG) and surgical ligation of CAF.

### 2.1 Surgical technique

**Fig. 3:**
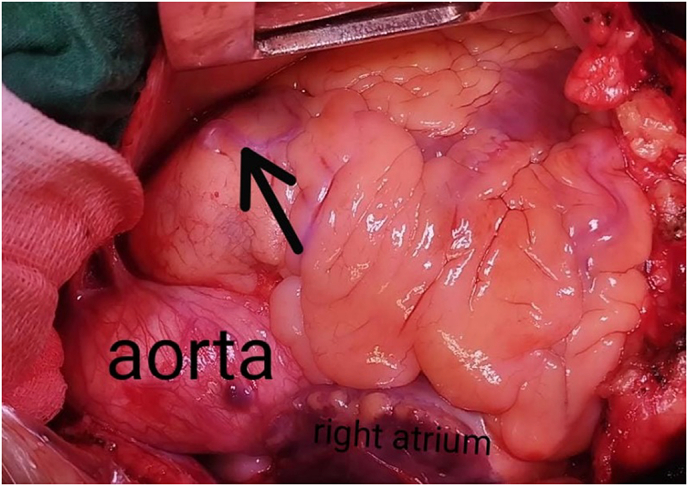
Intra-op photo showing epicardial CAF terminating in main PA.

**Fig. 4:**
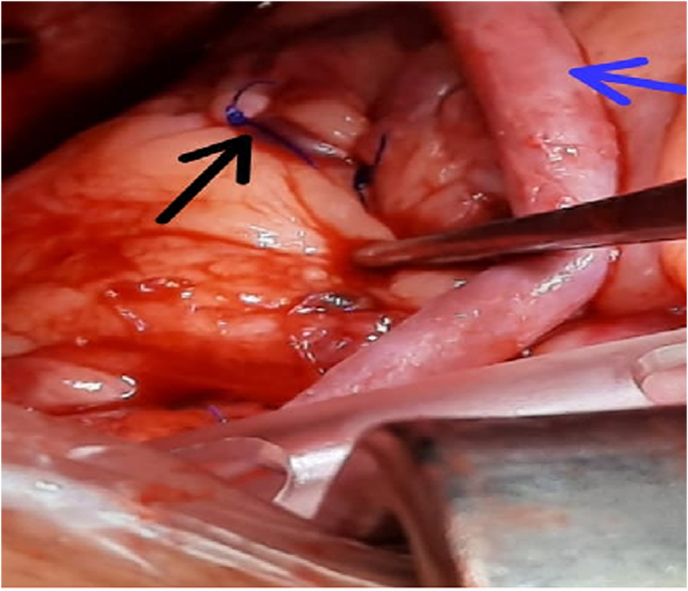
Intra-Op Photo showing epicardial ligation of fistula over PA with 4-0 proline (black arrow) and RSVG to 2nd Diagonal (blue arrow). (For interpretation of the references to colour in this figure legend, the reader is referred to the web version of this article.)

Under general anesthesia patient underwent full median sternotomy. After pericardiotomy, examination of the epicardium revealed fistula arising from the proximal LAD, running over anterior surface of heart, and draining into the main PA (Fig. 3). Cardiopulmonary bypass (CPB) was established via aorto-right atrial cannulation and cardiac arrest achieved via antegrade cardioplegia. Fistula was double ligated epicardially at the main PA with proline 4/0 suture (Fig. 4). Thereafter double vessel bypass was performed via left internal mammary artery to LAD and saphenous vein graft to 2nd diagonal. Patient made uneventful recovery in ICU and was discharged on 5th post-operative day.

Special attention was given towards myocardial protection. As soon as cardiopulmonary bypass (CPB) was established, cross clamp was applied across aorta and main PA followed by immediate antegrade root cardioplegia because after CPB there was high risk of blood flow from LAD to PA causing coronary steal from distal LAD. Cross clamping PA along with Aorta decreased the amount of cardioplegia shunting towards PA. Some cases may require retrograde cardioplegia for ideal myocardial protection and avoiding perioperative myocardial infarction.

## 3 Follow-up

Patient followed up in outpatient department 2 weeks after discharge and was doing well.

## 4 Discussion

CAF may be single or multiple in number. The commonest site of origin of CAF are right coronary artery (RCA) in 60 %, left anterior descending artery (LAD) 40 % and left circumflex artery (LCX) 18 % of cases [1]. More than one coronary arteries are involved in 5 % cases only [1]. The common draining sites of CAF include the right ventricle in 41 %, right atrium 26 %, pulmonary artery 17 % and coronary sinus 7 % of cases [5]. CAF usually leads to blood shunting from left to right side of heart.

Coronary to pulmonary artery fistula (CPAF) is an abnormal connection between the coronary artery and the pulmonary artery leading to blood shunting from left to right side throughout the cardiac cycle. In our case fistula was arising from the proximal LAD and draining into main pulmonary artery with left to right shunting of blood. Depending on the amount of shunting, consequences include pulmonary hypertension, rupture, and coronary steal phenomena. Other complications include endocarditis, early atherosclerosis, hemopericardium, and myocardial ischemia [5]. In our case the patient developed early atherosclerosis of the LAD artery at the site of origin of fistula.

Majority of the patients with CAF are asymptomatic as they have small and insignificant shunts [6]. However, the most common presenting symptom is angina [7]. Angina can be due to coronary artery disease (CAD) or coronary steal phenomena in the absence of CAD [7]. Other symptoms are consisted with congestive heart failure, rupture of fistula and endocarditis. The classical physical finding in some cases of CAF is a continuous murmur [7]. In our patient, this congenital fistula was likely small and asymptomatic initially, however with time he started symptoms of angina due to development of the significant CAD in LAD and coronary steal phenomena. There is also a possibility of fistula increased in size with time.

Investigation modalities include coronary angiography, echocardiography (ECHO), computed tomography, and cardiac MRI. Coronary angiography remains the gold standard for diagnosing CAF [8]. Coronary angiography identifies the origin of the fistula, its size and its draining chamber or vessel [2], [7]. It also shows the presence or absence of associated coronary artery disease, as was the case in our patient, however it is an invasive procedure and only gives a two-dimensional view of the anomaly. Non- invasive imaging techniques like Computed tomography (CT) and Echocardiography are becoming more common [2]. Multidetector computed tomography (MDCT) provides anatomical delineation, three dimensional reconstruction of fistula, its size and any associated aneurysm. MDCT is better than coronary angiography for its ability to show the fistula separately from the adjacent cardiovascular structures, along with any aneurysm or obstruction along its course. Other investigational modalities include transthoracic ECHO, transesophageal ECHO, and Cardiac Magnetic resonance imaging (MRI). ECHO identifies the severity of shunting, size of cardiac chambers and severity of pulmonary hypertension [2]. Coronary angiography, ECHO and MDCT was done in our case, besides diagnosing the condition and associated CAD, it also gives detailed information about the anatomy thus helping in peri-operative planning.

Treatment options of CAF include medical, percutaneous, and surgical approach [9]. The management of asymptomatic fistula diagnosed accidentally is controversial [1]. Conservative treatment is recommended for asymptomatic patients [3]. In asymptomatic patients' closure of fistula is only indicated in case of a large size fistula, development of aneurysm and increased degree of shunting causing hemodynamic overload on the heart [6]. In our case CAF ligation was done due to patient undergoing coronary artery bypass grafts, because we believe it is essential to ligate it otherwise it would result in coronary steal phenomenon leading to continued myocardial ischemia.

Surgical closure is done via median sternotomy with or without cardiopulmonary bypass. The surgical correction options for coronary artery fistulas include epicardial ligation, transection and closure of the drainage opening [3]. This also helps in taking care of any associated congenital defects or CAD in a patient.

In transcatheter closure different types of embolization materials are used, such as detachable balloons, stainless steel coils, platinum micro coils, controlled-release coils, regular stents, covered stents, and various chemicals [9]. Transcatheter closure is less invasive and less expensive [2]. It is associated with decreased mortality and morbidity and decreased recovery time as compared to surgical management provided the expertise is available [2], [9].

## 5 Conclusion

Our case is a good example of appropriate management of a rare congenital cardiac disease according to course of the disease. Surgical approach was chosen because patient became symptomatic after a period of 20 years and developed severe coronary artery disease in combination with CAF. Myocardial protection needs special attention in these cases as initiation of CPB can lead to coronary steal phenomena and hence perioperative myocardial infarction. Early CAF ligation is necessary in its natural course to avoid catastrophic complications like cardiac arrest and cardiac tamponade.

## Source of funding

Nothing to declare.

## Consent

Written informed consent was obtained from the patient for publication of this case report and accompanying images. A copy of the written consent is available for review by the Editor-in-Chief of this journal on request.

## Ethical approval

Exemption from hospital Ethical Review Committee.

## Author contribution

Mian Mustafa Kamal: Case report Concept and Design.

Abdul Ahad Sohail: Data Collection and writing of manuscript.

Rita Sundardas: Data Collection and writing of manuscript.

Majid Usman: Analysis and Interpretation and Critical Review.

Sara Iqbal: Analysis and Interpretation and Critical Review.

Fateh Ali Tipu: Analysis and Interpretation and Critical Review.

Saulat Fatimi: Analysis and Interpretation and Critical Review.

## Registration of research studies

Not applicable.

## Guarantor

Mian Mustafa kamal.

Saulat Hasnain Fatimi.

## Provenance and peer review

Not commissioned, externally peer-reviewed.

## Declaration of competing interest

Nothing to declare.
